# Real-Time (Vision-Based) Road Sign Recognition Using an Artificial Neural Network

**DOI:** 10.3390/s17040853

**Published:** 2017-04-13

**Authors:** Kh Tohidul Islam, Ram Gopal Raj

**Affiliations:** Department of Artificial Intelligence, Faculty of Computer Science & Information Technology, University of Malaya, Kuala Lumpur 50603, Malaysia; kh.tohidulislam@gmail.com

**Keywords:** intelligent transportation system, artificial intelligence, computer vision, road and traffic sign recognition

## Abstract

Road sign recognition is a driver support function that can be used to notify and warn the driver by showing the restrictions that may be effective on the current stretch of road. Examples for such regulations are ‘traffic light ahead’ or ‘pedestrian crossing’ indications. The present investigation targets the recognition of Malaysian road and traffic signs in real-time. Real-time video is taken by a digital camera from a moving vehicle and real world road signs are then extracted using vision-only information. The system is based on two stages, one performs the detection and another one is for recognition. In the first stage, a hybrid color segmentation algorithm has been developed and tested. In the second stage, an introduced robust custom feature extraction method is used for the first time in a road sign recognition approach. Finally, a multilayer artificial neural network (ANN) has been created to recognize and interpret various road signs. It is robust because it has been tested on both standard and non-standard road signs with significant recognition accuracy. This proposed system achieved an average of 99.90% accuracy with 99.90% of sensitivity, 99.90% of specificity, 99.90% of f-measure, and 0.001 of false positive rate (FPR) with 0.3 s computational time. This low FPR can increase the system stability and dependability in real-time applications.

## 1. Introduction

A road sign recognition system can technically be developed as part of an intelligent transportation system that can continuously monitor the driver, the vehicle, and the road in order, for example, to inform the driver in time about upcoming decision points regarding navigation and potentially risky traffic situations. Road sign detection and recognition [1] is an essential part of the Autonomous Intelligence Vehicle Design (AIVD) [2]. It is widely used for intelligent driving assistance [3], self-directed vehicles, traffic rules and regulation awareness, disabled (blind) pedestrian awareness and so on. On the other hand, road sign detection and recognition can also be a part of self-driving car [4] technology to determine the road-traffic environment in real-time.

Detection and recognition is one of the most challenging tasks in the field of computer vision [5] and digital image processing to detect a specific object in a real-time environment [6]. Researchers are paying more attention in intelligent transportation systems [7]. Some of them have successfully implemented road sign recognition methods to detect and recognize red-colored road signs [8] only or single classes of road signs [9,10,11,12,13], and some of them have used specific country road signs [7,14,15,16]. In this field, a group of researchers has already shown distinguished performance based on annotated road signs [14,16,17]. Overall, for a standard road sign recognition approach, further improvements are needed.

The aim of this research was to overcome the current limitations of road sign recognition such as single-color or single-class and specific country’s road signs. It is quite challenging to fulfill the road sign recognition task in a real-time changing environment. Among the many issues that must be addressed are the low light conditions, fading of signs, and most importantly, non-standard signs, which also degrade the performance of the road sign recognition system.

**Figure 1:**
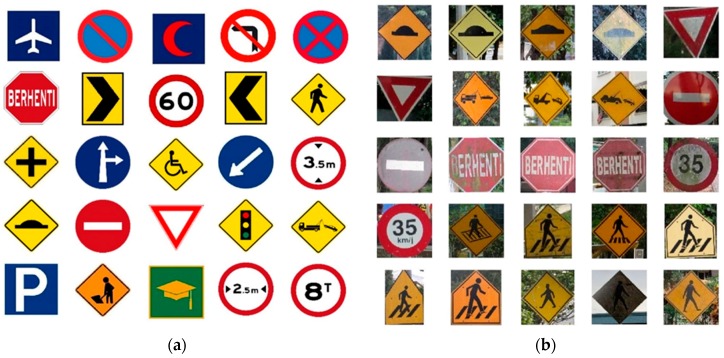
Different road signs in Malaysia. (**a**) Standard Malaysian road signs as adapted from ARHAN TEKNIK (JALAN) 2A/85; (**b**) Non-standard road signs appearing in the Malaysian highway system.

Road signs which are designed for human eyes, are easily detectable and recognizable [16], even with significant variations, but for a computer vision system, small variations cannot be adjusted automatically, so it needs proper guidance. Standard color and shape are the main properties of standard road signs [18]. Though the road sign has state of the art, various natural issues and human errors cause variations in color, shape, or both. For instance, multiple non-standard road signs may be found on Malaysian highways, as seen in Figure 1b.

This paper focused on the standard and non-standard road sign detection and recognition. Accuracy is a key consideration, because one misclassified or undetected sign could have an adverse impact on the driver. The main objective of this research is to develop a robust hybrid algorithm that can be used in a wide range, to evaluate the system performance with other existing methods and eventually to evaluate the classification algorithm performance. The proposed method consists of the following two stages: (1) detection and (2) recognition.

Detection is performed by using video frame segmentation and hybrid color segmentation algorithms. This hybrid color segmentation algorithm contains a RGB histogram equalization, RGB color segmentation, modified grey scale segmentation, binary image segmentation, and a shape matching algorithm. A RGB color segmentation algorithm is used for subtracting red (R), green (G) and blue (B) components of the input image. In the next step, a RGB to grayscale converter is used to convert the subtracted image into a grayscale image. Then, a 2-dimensional 3-by-3 median filter is used to remove existing noise from the grayscale images. Next, it replaces all the input image pixels’ value with luminance that is greater than 0.18 to 1 as a white pixel, and all others pixels are replaced to 0 as a black pixel. From this process, a grayscale image is converted into a binary image. A threshold level of 0.18 is used for this conversion process, because it gives the best performance for this system. After that conversion, the first step is removing all the small objects from the binary images which contain less than 300 pixels and then labeling all connected components using 8-connected objects. The next step is measuring of the image region properties, and how many candidates are available on that binary image is found. This is the target candidate to identify as a road sign. Then from the target candidate, the algorithm determines the position (X, Y-coordinate), height (H), and width (W) of every single object accordingly. For the candidate selection, candidates which have a height (H) and width (W) ratio close to 1, are considered as a target candidate. Based on the selected candidates’ properties, the sign image is cropped from the original RGB input frame. That input frame is also a high resolution RGB image with target objects. Here, the detected target road sign contains enough pixel information because it is extracted from an original RGB input frame. Initially, that detected road sign image is resized to 128-by-128 pixels. Then that RGB image has been converted into a grayscale image and existing noise removed by using 3-by-3 2-dimensional median filter. Then, the grayscale image is converted into a binary image with an average grayscale histogram level. All these algorithms are tested using thousands of images. The hybrid color segmentation algorithm has eventually been chosen for this proposed system as it shows the best performance for detection of road signs. Finally, a robust custom feature extraction method has been introduced for the first time in the road sign recognition system to extract multiple features from a single image.

Training images are collected by acquiring appropriate frames from video sequences, which were captured on different roads and highways in Malaysia in a real-time environment. For the recognition, an artificial neural network is implemented by using the Neural Network Pattern Recognition Tool with MATLAB. The standard network that is used for pattern recognition, is a two-layer feedforward network, with a sigmoid transfer function in the hidden layer, and a softmax transfer function in the output layer. Real-time video frames goes through this network. However, signs which may completely be obscured by other vehicles or trees may not be recognized, although the system recognizes and interprets various standard and non-standard road signs using vision-only information. It has reached an exceptionally high recognition accuracy.

In this work, in order to achieve robust, fast detection and recognition of road signs, a hybrid color segmentation algorithm with a robust custom feature extraction method has been proposed. This feature extraction method achieves robustness in improving the computation efficiency. Further, in order to reduce the classification time, ANN-based classification which has been selected with comparing the classification performance among with other classifiers, is implemented. Experimental results show that this work achieves robust road sign recognition in comparison to the other existing methods, and achieves high recognition accuracy with low false positive rate (FPR). This low FPR can increase the system stability and reliability for real-time applications. Multiple robustness testing results have indicated that this proposed system is able to achieve promising performance, even in adverse conditions.

## 2. Related Work

Various road sign detection and recognition methods and algorithms have been developed [19,20,21,22,23,24]. All researchers are implementing their methods to achieve a common goal [25]. Some researchers have done the detection [26,27,28] part, some are tracking the detection and a few have described effective recognition parts [29,30]. According to Paclík et al. [31], an automated traffic sign detection system was introduced for the first time in Japan in 1984. In the field of road sign recognition, the most common approach has two (2) main stages which are firstly detection and secondly recognition. The detection stage works to identify the proper region of interest (ROI) and color segmentation algorithms are mostly used. This detection stage is performed and followed by some form of shape detection and recognition. In the recognition stage, detected candidates are either rejected or identified with some recognition methods, for example, shape matching [32] and some other form of classifiers such as ANN [15], support vector machine (SVM) [7,33,34], clustering [35], and fuzzy logic [9].

Mostly, the color information method is used for image segmentation to make up the majority of system’s detection part [36,37,38,39,40,41,42,43]. A color matching method was introduced by De La Escalera et al. [44] where they used it to look for patterns in a specific correspondence relationship to rectangular, triangular and some circular signs. However their proposed method faced some difficulties regarding different road signs with the same shape. For sign recognition, a physics-based [45] method was used, but it needed to keep in memory changes in the parameter model to accommodate the natural variation in illumination [46]. A neural network [47] was used to recognize road and traffic signs for an intelligent driving assistance system, but this system showed some contradictory road and traffic sign pattern results with complex image backgrounds. A real-time road and traffic sign detection plus recognition system were developed by Ruta et al. [48] to perform the recognition from a video using class-specific discriminative features. An automatic colored traffic sign detection system [49] was developed by using optoelectronic correlation architectures. A real-time road and traffic sign recognition system were introduced by Deshmukh et al. [50] which was based on color image segmentation. They used a segmentation technique which was more difficult because the system had been developed in C language that was not so strong in comparison to MATLAB or OpenCV. Therefore, it can be concluded that the main difficulties of color-based road sign detection and recognition systems are illumination, adverse weather conditions and poor lighting conditions.

The Optical Character Recognition (OCR) [51] tool “Tesseract” was used to detect text in road and traffic signs. The results showed a higher accuracy compared to the HOG-SVMs system. A unique system for the automated detection and recognition of text in road traffic signs was proposed by Greenhalgh et al. [52]. A half structure was employed to outline search regions inside the image, within which traffic sign candidates were found. Maximally stable extremal regions (MSERs) and HUE, saturation and color thresholding area units were used to find an oversized range of candidates, after that those units were reduced by applying constraints supported by temporal and structural data. The strategy was relatively evaluated and it achieved an overall F-measure of 0.87.

Visual sign information extraction and recognition remain challenging due to the uncontrolled lightening conditions, occlusion, and variations in shape, size, and color [53]. Gil-Jimenez et al. [54] introduced a novel algorithm for shape classification which was based on the support vector machine (SVM) and the FFT of the signature of the blob. Khan et al. [32] investigated image segmentation and joint transform correlation (JTC) with the integration of shape analysis for a road sign recognition method. Their experimental results on real-life images showed a high success rate and a very low false hit rate. For the traffic sign detection, a hybrid active contour (HAC) algorithm was proposed by Ai et al. [55]. It dealt with a location probability distribution function (PDF), statistical color model (SCM), and global curve length and it was further improved by a new geometry-preserving active polygon (GPAP) model [56]. A video-based detection and classification of traffic signs which were based on color and shape benchmark, was investigated by Balali et al. [57]. They also introduced a roadway inventory management system based on the detection and classification of traffic signs from Google Street View images [58]. Chen et al. [59] presented a new traffic sign detection method by combining both the AdaBoost algorithm and support vector regression (SVR) which achieved fast and accurate detection.

From the related work, road sign colors represent the key information for drivers. Color is a significant source of information for the detection and recognition of road and traffic signs. As their colors are characteristic hallmarks of road and traffic signs, color can simplify this process. An important part of any color-based detection system is “color space conversion”, which converts the RGB image into other forms that simplify the detection process. This means that color space conversion separates the brightness of color information by converting RGB color space to another color space. This gives a good detection capability depending on the color of the tail. There are many color spaces available in the related works, namely, HIS [60], HBS, HSV [61], IHLS [62], L*a*b* [40] color system YIQ [60] and YUV [63]. Saturation color systems are mostly used in the detection of road signs, but the other color systems are also used for this task.

Various techniques are commonly used in the recognition of road signs as presented above. Some of those techniques may be combined with others to produce a hybrid recognition system. In comparison to the recognition based on color and shape, this approach may have several limitations. Gaps in the color-based recognition, such as those caused by weather conditions and faded road signs can be offset by using shape-based recognition which may give more superior performance. For color-based recognition, most approaches can work considerably faster in indexing color. Although the indexing color can segment an image when the sign of the road is slightly inclined or partially occluded, its calculation time increases sharply in a complex background. Therefore, color indexing is not ideal for implementation in real time applications. Color thresholding, on the other hand, may not be robust when the weather conditions are bad or a sign is faded.

For a Malaysian road and traffic sign recognition system, Wali et al. [8] developed a color segmentation algorithm with SVMs and their system performance was 95.71%. This is not sufficient for a complete stable system, so it needs further research to implement a stable version of their road and traffic sign recognition system.

After surveying different research works, the objective of the proposed system is to present a fast and robust system for road sign recognition which is a real-time vision-based. For the first time in a road sign recognition system, a robust custom feature extraction method is introduced to extract multiple features from a single input image. In this proposed approach, for reducing the processing time, a hybrid segmentation algorithm with shape measurement-based detection, and an ANN with custom features extraction are used to recognize road signs that can account for multiple variations in road signs. This hybrid segmentation algorithm is used to make this detection and recognition more robust to changes in illumination.

## 3. Traffic Sign Detection and Recognition System

### 3.1. System Overview

**Figure 2:**
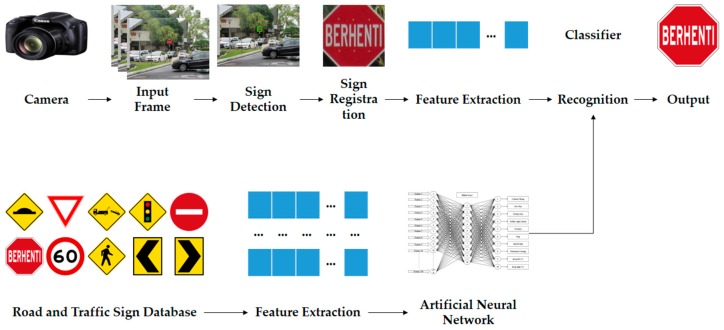
Road and Traffic Sign Recognition System Architecture.

Numerous real-world computer vision applications which have been developed require the accurate detection of target object from video sequences. Road sign recognition is one of the more challenging examples of real-world computer vision applications. Because of the high industrial relevance, many approaches for traffic sign detection and recognition have been proposed. Any proposed road sign recognition process should be able to work in the following two modes: the first mode is detection mode and it works as a primary stage to collect images from real-time road environments by capturing video from a moving vehicle. This video is segmented frame by frame and then goes through a hybrid segmentation algorithm to identify the road and traffic sign candidates. This hybrid color segmentation algorithm contains a RGB histogram equalization, RGB color segmentation, modified grey scale segmentation, binary image segmentation and shape matching algorithm. Histogram equalization improves the color constancy of all red, green, and blue regions, and RGB color segmentation extracts the red (R) region, green (G) region, and blue (B) region. If the road sign candidate is greater than zero (>0), that specific candidate saves as like an input image to an image database and that is an RGB image. The second mode is classification, which works with a robust custom feature extraction method and an artificial neural network (ANN) which is designed to train, test and validate the data. This robust custom features extraction process is based on a size- independent method and obtains 278 features for each road sign. The overall proposed system architecture is illustrated in Figure 2.

### 3.2. Image Acquisition

The sample images are collected from a reasonable quality onboard camera (Power Shot SX530 HS, Canon, Tokyo, Japan) which is placed inside a moving vehicle. Training images are collected by acquiring appropriate frames from video sequences captured on different roads and highways in Malaysia in a real-time environment from 8:00 a.m. to 6:00 p.m. As Malaysian cars drive on the left side of the road, the camera is placed on the left side of the dashboard to capture the traffic signs on the left side. A video frame segmentation algorithm is used to segment the video frame by frame with 1 second interval for further analysis. The aim of this is to create a real-time database of road sign images under different conditions which are summarized in Table 1.

### 3.3. Feature Extraction

For the first time in a road sign recognition system, a robust custom feature extraction method is introduced to extract multiple features from a single input image. This robust custom feature extraction processes is based on a size independent method and obtains 278 features for road signs. Initially, a 128-by-128 pixels binary image is converted to a 128-by-128 binary matrix. The total pixel value is 128-by-128 = 16384 pixels. A matrix ‘A’ represents a 128-by-128 binary matrix shown in Equation (3) where *m* = 128 and *n* = 128.

(1) $$A=\left[\begin{array}{cccc} a_{11} & a_{12} & \ldots \ldots & a_{1n}\\ a_{21} & a_{22} & \ldots \ldots & a_{2n}\\ \vdots & \vdots & \ddots & \vdots \\ a_{m1} & a_{m2} & \ldots \ldots & a_{mn} \end{array}\right]$$

True pixels is the summation of all white pixels using Equation (2):

(2) $$True\;pixel=\overset{128}{\underset{m=1}{\sum }}\overset{128}{\underset{n=1}{\sum }}A_{mn}$$

**Figure 3:**
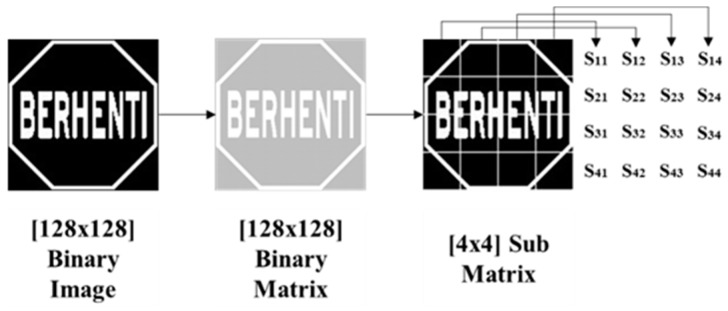
Subregion segmentation.

Now, this original matrix A is divided into a submatrix which is S = 4-by-4 matrix by Equation (3) where 1≤ *m* ≥4 and 1≤ *n* ≥ 4 and is shown in Figure 3. This 4-by-4 sub matrix pixel value needs to be stored in the system database for further processing.

(3) $$S=\left[\begin{array}{cccc} s_{11} & s_{12} & \ldots \ldots & s_{1n}\\ s_{21} & s_{22} & \ldots \ldots & s_{2n}\\ \vdots & \vdots & \ddots & \vdots \\ s_{m1} & s_{m2} & \ldots \ldots & s_{mn} \end{array}\right]$$

In this submatrix, each element is the summation of the original matrix’s array elements which are defined by Equation (4). The summation conditions are given in Table 2.

(4) $$s=\left[\begin{array}{cccc} s_{11} & s_{12} & \ldots \ldots & s_{14}\\ s_{21} & s_{22} & \ldots \ldots & s_{24}\\ \vdots & \vdots & \ddots & \vdots \\ s_{41} & s_{42} & \ldots \ldots & s_{44} \end{array}\right]$$

**Figure 4:**
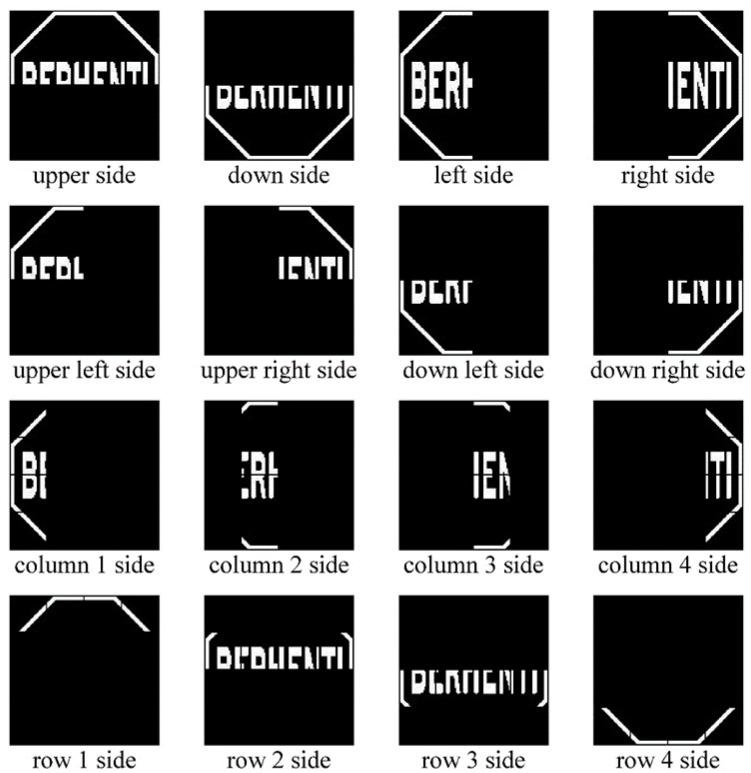
Image subregion feature extraction.

Custom features extraction is performed by dividing the 128-by128 binary matrix into multiple submatrices. Road signs are divided into multiple areas such as upper side, down side, left side, right side, upper left side, upper right side, down left side, down right side, four columns side and four rows side which are shown in Figure 4. Then the feature extraction algorithm calculates all existing white pixel values in that area.

Equations (5) and (6) represent the row and the column vectors, respectively, from submatrix *S*:

(5) $$RV=\overset{4}{\underset{m=1}{\sum }}S_{mn};m=1,\ldots ,\ldots ,4$$

(6) $$RV=\overset{4}{\underset{n=1}{\sum }}S_{mn};n=1,\ldots ,\ldots ,4$$

**Figure 5:**
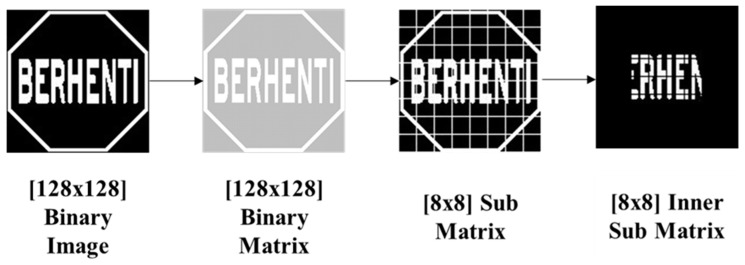
Sub matrix and inner sub matrix for pictogram analysis.

**Figure 6:**
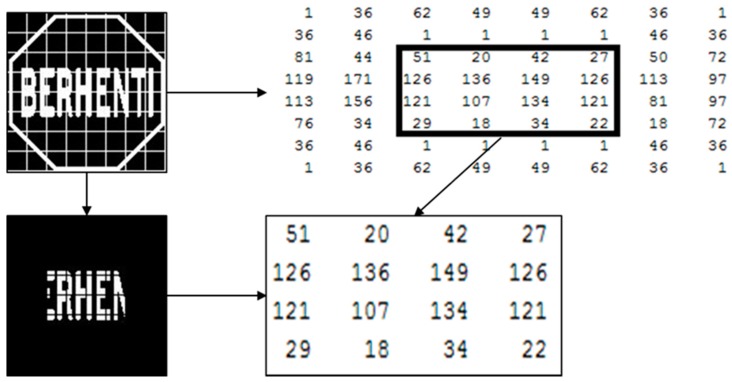
An example of the pictogram feature extraction of a STOP sign.

**Figure 7:**
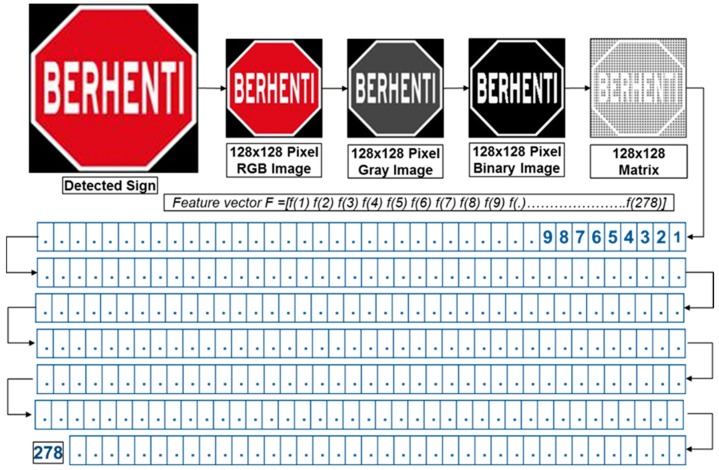
An overview of the feature extraction step.

**Figure 8:**
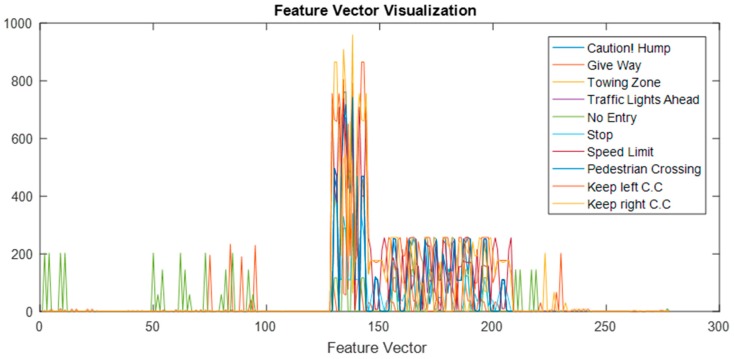
Feature vector visualization.

Figure 5 shows an 8-by-8 inner sub matrix for pictogram pattern analysis. An example pictogram pattern analysis of a STOP sign is shown in Figure 6. The feature extraction overview in Figure 7 and Figure 8 shows the features vector visualization.

From the 8-by-8 binary matrix, a region of interest (ROI) is extracted for pictogram feature analysis which is also shown in Figure 6.

The custom feature extraction algorithm works in several steps. Every new step is started after finishing the previous step. The process can be represented by the following steps:

1. Read 128-by-128 binary image as A = 128-by-128 matrix.
2. Compute Total Pixels, total_pixels as 128x128 = 16384.
3. Compute True Pixels, true_pixels as the sum of all white pixels of A.
4. Compute 4-by-4 sub matrix of A as sub = 4-by-4 sub matrix of A.
5. Compute 8-by-8 sub matrix of A as S = 8-by-8 sub matrix of A.
6. Compute 4-by-4 sub matrices Rows and Columns as row1 = sub (1) + sub (5) + sub (9) + sub (13); row2 = sub (2) + sub (6) + sub (10) + sub (14); row3 = sub (3) + sub (7) + sub (11) + sub (15); row4 = sub (4) + sub (8) + sub (12) + sub (16); column1 = sub (1) + sub (2) + sub (3) + sub (4); column2 = sub (5) + sub (6) + sub (7) + sub (8); column3 = sub (9) + sub (10) + sub (11) + sub (12); column4 = sub (13) + sub (14) + sub (15) + sub (16);
7. Compute 8-by-8 sub matrices sub Rows and sub Columns as srow1 = s(19) + s(27) + s(35) + s(43); srow2 = s(20) + s(28) + s(36) + s(44); srow3 = s(21) + s(29) + s(37) + s(45); srow4 = s(22) + s(30) + s(38) + s(46); scolumn1 = s (19) + s (20) + s (21) + s (22); scolumn2 = s (27) + s (28) + s (29) +s (30); scolumn3 = s (35) + s (36) + s (37) + s (38); scolumn4 = s (43) + s (44) + s (45) + s (46);
8. Compute Sum of Upper Pixels as up = row1 + row2;
9. Compute Sum of Dows Pixels as dp = row3 + row4;
10. Compute Sum of Left Pixels as lp = column1 + column2;
11. Compute Sum of Right Pixels as rp = column3 + column4;
12. Compute Sum of Upper Left Pixels as ulp = sub (1) + sub (5) + sub (2) + sub (6);
13. Compute Sum of Upper Right Left Pixels as urp = sub (9) + sub (13) + sub (10) + sub (14);
14. Compute Sum of Down Left Pixels as dlp = sub (3) + sub (7) + sub (4) + sub (8);
15. Compute Sum of Down Right Pixels as drp = sub (11) + sub (15) + sub (12) + sub (16);
16. Compute Sum of Upper Left Upper Pixels as ulup = sub (1) + sub (5);
17. Compute Sum of Upper Left Down Pixels as uldp = sub (2) + sub (6);
18. Compute Sum of Upper Left-Left Pixels as ullp = sub (1) + sub (2);
19. Compute Sum of Upper Left Right Pixels as ulrp = sub (5) + sub (6);
20. Compute Sum of Upper Right Upper Pixels as urup = sub (9) + sub (13);
21. Compute Sum of Upper Right Down Pixels as urdp = sub (10) + sub (14);
22. Compute Sum of Upper Right Left Pixels as urlp = sub (9) + sub (10);
23. Compute Sum of Upper Right-Right Pixels as urrp = sub (13) + sub (14);
24. Compute Sum of Down Left Upper Pixels as dlup = sub (3) + sub (7);
25. Compute Sum of Down Left Down Pixels as dldp = sub (4) + sub (8);
26. Compute Sum of Down Left-Left Pixels as dllp = sub (3) + sub (4);
27. Compute Sum of Down Left Right Pixels as dlrp = sub (7) + sub (8);
28. Compute Sum of Down Right Upper Pixels as drup = sub (11) + sub (15);
29. Compute Sum of Down Right Down Pixels as drdp = sub (12) + sub (16);
30. Compute Sum of Down Right Left Pixels as drlp = sub (11) + sub (12);
31. Compute Sum of Down Right-Right Pixels as drrp = sub (15) + sub (16);
32. Compute average 8-by-8 sub matrices as av = (srow1 + srow2 + srow3 + srow4)/16.

The algorithm extracts all the required information by following these 32 steps. This information was used for features extraction. Features 1–12 were defined based on 4-by-4 sub matrix rows. Feature 1 will be the ratio between the first row and the second row and Feature 2 will be the ratio between the second row and the first row. This process will continue to the fourth row, and Feature 12 will be the ratio between the fourth row and third row. The algorithm is as follows:

*f1 = row1/row2; f2 = row2/row1; f3 = row1/row3; f4 = row3/row1; f5 = row1/row4; f6 = row4/row1; f7 = row2/row3; f8 = row3/row2; f9 = row2/row4; f10 = row4/row2; f11 = row3/row4; f12 = row4/row3;*

Features 13–24 were defined based on 4-by-4 sub matrices columns. Features 13–24 will be same as the Features 1–12. The only difference is that Features 1–12 depend upon rows and Features 13–24 depend upon columns. Feature 13 will be the ratio between the first column and the second column. Feature 24 will be the ratio between the fourth column and the third column. The algorithm is as follows:

*f13 = column1/column2; f14 = column2/column1; f15 = column1/column3; f16 = column3/column1; f17 = column1/column4; f18 = column4/column1; f19 = column2/column3; f20 = column3/column2; f21 = column2/column4; f22 = column4/column2; f23 = column3/column4; f24 = column4/column3;*

Features 25–36 were defined based on Upper Pixels, Down Pixels, Left Pixels, and Right pixels. The 128-by-128 matrix is divided up into horizontally and vertically as upper pixels, down pixels, left pixels, and right pixels. Feature 25 will be the ratio between the upper pixels’ value and down pixels’ value. Feature 36 will be the ratio between the right pixels’ value and left pixels’ value. The algorithm is as follows:

*f25 = up/dp; f26 = dp/up; f27 = up/lp; f28 = lp/up; f29 = up/rp; f30 = rp/up; f31 = dp/lp; f32 = lp/dp; f33 = dp/rp; f34 = rp/dp; f35 = lp/rp; f36 = rp/lp;*

Features 37–48 were defined based on Upper Left Pixels, Upper Right Pixels, Down Left Pixels, and Down Right Pixels which are shown in Figure 4 (2nd row). The 128-by-128 matrix is divided up into four parts which are Upper Left Pixels part is the left top part, Upper Right Pixels part is the top right part, Down Left Pixels part is the bottom left part, and Down Right Pixels part is the bottom right part. Feature 37 will be the ratio between the upper left pixels’ value and the upper right pixels’ value. Feature 48 will be the ratio between the down right pixels’ value and the down left pixels’ value. The algorithm is as follows:

*f37 = ulp/urp; f38 = urp/ulp; f39 = ulp/dlp; f40 = dlp/ulp; f41 = ulp/drp; f42 = drp/ulp; f43 = urp/dlp; f44 = dlp/urp; f45 = urp/drp; f46 = drp/urp; f47 = dlp/drp; f48 = drp/dlp;*

The Upper Left Pixels part is divided into four subparts which are Upper Left Upper Pixels, Upper Left Down Pixels, Upper Left-Left Pixels, and Upper Left Right Pixels. Features 49–60 were defined based on those subparts. Feature 49 will be the ratio between the upper left upper pixels’ values and the upper left down pixels’ value. Feature 60 will be the ratio between the upper left right pixels’ value and the upper left-left pixels’ value. The algorithm is as follows:

*f49 = ulup/uldp; f50 = uldp/ulup; f51 = ulup/ullp; f52 = ullp/ulup; f53 = ulup/ulrp; f54 = ulrp/ulup; f55 = uldp/ullp; f56 = ullp/uldp; f57 = uldp/ulrp; f58 = ulrp/uldp; f59 = ullp/ulrp; f60 = ulrp/ullp;*

Upper Right Pixels part is divided into four subparts which are Upper Right Upper Pixels, Upper Right Down Pixels, Upper Right Left Pixels, and Upper Right Right-Pixels. Features 61–72 were defined based on those subparts. The process is same as earlier, whereby Feature 61 will be the ratio between the upper right upper pixels’ value and the upper right down pixels’ value. Feature 72 will be the ratio between the upper right-right pixels’ value and the upper right left pixels’ value. The algorithm is as follows:

*f61 = urup/urdp; f62 = urdp/urup; f63 = urup/urlp; f64 = urlp/urup; f65 = urup/urrp; f66 = urrp/urup; f67 = urdp/urlp; f68 = urlp/urdp; f69 = urdp/urrp; f70 = urrp/urdp; f71 = urlp/urrp; f72 = urrp/urlp;*

Down Left Pixels part is divided into four subparts which are Down Left Upper Pixels, Down Left Down Pixels, Down Left-Left Pixels, and Down Left Right Pixels and Features 73–84 were defined based on those subparts. Feature 73 will be the ratio between the down left upper pixels’ value and the down left down pixels’ value. Feature 84 will be the ratio between the down left right pixels’ value and the down left-left pixels’ value. The algorithm is as follows:

*f73 = dlup/dldp; f74 = dldp/dlup; f75 = dlup/dllp; f76 = dllp/dlup; f77 = dlup/dlrp; f78 = dlrp/dlup; f79 = dldp/dllp; f80 = dllp/dldp; f81 = dldp/dlrp; f82 = dlrp/dldp; f83 = dllp/dlrp; f84 = dlrp/dllp;*

Down Right Pixels part is divided into four subparts which are Down Right Upper Pixels, Down Right Down Pixels, Down Right Left Pixels, and Down Right-Right Pixels and Features 85–96 were defined based on those subparts. Feature 85 will be the ratio between the down right upper pixels’ value and the down right down pixels’ value. Feature 96 will be the ratio between the down right-right pixels’ value and the down right left pixels’ value. The algorithm is as follows:

*f85 = drup/drdp; f86 = drdp/drup; f87 = drup/drlp; f88 = drlp/drup; f89 = drup/drrp; f90 = drrp/drup; f91 = drdp/drlp; f92 = drlp/drdp; f93 = drdp/drrp; f94 = drrp/drdp; f95 = drlp/drrp; f96 = drrp/drlp;*

Features 97–128 were defined based on the true pixel value. A true pixel value is the sum of all white pixels’ value of the image region. All the variables which are declared for defining of Feature 1 to Feature 96, will be divided by the true pixel value. Feature 97 will be the ratio between the first variable (row1) and the true pixel value. Feature 128 will be the ratio between the last variable (down right-right pixels) and the true pixel value.

Features 129–144 were defined based on the individual elements of the 4-by-4 sub matrix which is shown in Figure 3. Feature 129 will be the first element of the 4-by-4 submatrix which is S_11_. Feature 144, which is S_44_, will be the 16th element of the 4-by-4 sub matrix because a 4-by-4 submatrix has 16 elements.

Features 145–208 were defined based on the individual elements of the 8-by-8 sub matrix which is shown in Figure 5. Feature 145 will be the first element of the 8-by-8 matrix. Feature 208 will be the 64th element of the 8-by-8 sub matrix because a 8-by-8 sub matrix has 64 elements.

Features 209–232 were defined based on the 8-by-8 sub matrix’s 4-by-4 inner submatrix which is shown in Figure 6. The inner submatrix represents the pictogram pixels’ value of a traffic sign. Feature 209 will be the ratio between the inner submatrix’s first row and second row and Feature 232 will be the ratio between the inner submatrix’s forth column and third column. The algorithm is as follows:

*f209 = srow1/srow2; f210 = srow2/srow1; f211 = srow1/srow3; f212 = srow3/srow1; f213 = srow1/srow4; f214 = srow4/srow1; f215 = srow2/srow3; f216 = srow3/srow2; f217 = srow2/srow4; f218 = srow4/srow2; f219 = srow3/srow4; f220 = srow4/srow3; f221 = scolumn1/scolumn2; f222 = scolumn2/scolumn1; f223 = scolumn1/scolumn3; f224 = scolumn3/scolumn1; f225 = scolumn1/scolumn4; f226 = scolumn4/scolumn1; f227 = scolumn2/scolumn3; f228 = scolumn3/scolumn2; f229 = scolumn2/scolumn4; f230 = scolumn4/scolumn2; f231 = scolumn3/scolumn4; f232 = scolumn4/scolumn3;*

Features 233–244 were defined based on the ratio between every element of the inner submatrix. Feature 233 will be the ratio between the first element and the second element of the inner submatrix. Feature 244 will be the ratio between the last element and the second-last element of that inner sub- matrix. The algorithm is as follows:

*f233 = sub(6)/sub(7); f234 = sub(7)/sub(6); f235 = sub(6)/sub(10); f236 = sub(10)/sub(6); f237 = sub(6)/sub(11); f238 = sub(11)/sub(6); f239 = sub(7)/sub(10); f240 = sub(10)/sub(7); f241 = sub(7)/sub(11); f242 = sub(11)/sub(7); f243 = sub(10)/sub(11); f244 = sub(11)/sub(10);*

Features 245–260 were defined based on the ratio between every element of the inner submatrix to the true pixels’ value. Feature 245 will be the ratio between the first element of that matrix and the true pixels’ value and Feature 260 will be the ratio between the last element of that matrix and the true pixels’ value. The algorithm is as follows:

*f245 = sub(1)/tp; f246 = sub(2)/tp; f247 = sub(3)/tp; f248 = sub(4)/tp; f249 = sub(5)/tp; f250 = sub(6)/tp; f251 = sub(7)/tp; f252 = sub(8)/tp; f253 = sub(9)/tp; f254 = sub(10)/tp; f255 = sub(11)/tp; f256 = sub(12)/tp; f257 = sub(13)/tp; f258 = sub(14)/tp; f259 = sub(15)/tp; f260 = sub(16)/tp;*

Features 261–276 were defined based on the average value of the 8-by-8 submatrix and every element of the 4-by-4 inner submatrix. Feature 261 will be the ratio between the first element of the inner submatrix and the average value of the 8-by-8 submatrix. Feature 276 will be the ratio between the last element of the inner submatrix and the average value of the 8-by-8 sub matrix. The algorithm is as follows:

*f261 = s(19)/av; f262 = s(20)/av; f263 = s(21)/av; f264 = s(22)/av; f265 = s(27)/av; f266 = s(28)/av; f267 = s(29)/av; f268 = s(30)/av; f269 = s(35)/av; f270 = s(36)/av; f271 = s(37)/av; f272 = s(38)/av; f273 = s(43)/av; f274 = s(44)/av; f275 = s(45)/av; f276 = s(46)/av;*

The final two features are based on the ratio between the total pixels’ value and the true pixels’ value. Feature 277 will be the ratio between the total pixels’ value and the true pixels’ value and Feature 278 will be the ratio between the true pixels’ value and the total pixels’ value. The algorithm is as follows:

*f277 = total_pixels/tp; f278 = tp/total_pixels;*

The final feature extraction process provides the 278 feature values. All these feature values are needed to build a feature matrix to identify the road signs. The 1-by-278 feature matrix is determined as follows:

F = [f1 f2 f3 f4 f5 f6 f7 f8 f9 f10 f11 f12 f13 f14 f15 f16 f17 f18 f19 f20 f21 f22 f23 f24 f25 f26 f27 f28 f29 f30 f31 f32 f33 f34 f35 f36 f37 f38 f39 f40 f41 f42 f43 f44 f45 f46 f47 f48 f49 f50 f51 f52 f53 f54 f55 f56 f57 f58 f59 f60 f61 f62 f63 f64 f65 f66 f67 f68 f69 f70 f71 f72 f73 f74 f75 f76 f77 f78 f79 f80 f81 f82 f83 f84 f85 f86 f87 f88 f89 f90 f91 f92 f93 f94 f95 f96 f97 f98 f99 f100 f101 f102 f103 f104 f105 f106 f107 f108 f109 f110 f111 f112 f113 f114 f115 f116 f117 f118 f119 f120 f121 f122 f123 f124 f125 f126 f127 f128 f129 f130 f131 f132 f133 f134 f135 f136 f137 f138 f149 f140 f141 f142 f143 f144 f145 f146 f147 f148 f149 f150 f151 f152 f153 f154 f155 f156 f157 f158 f159 f160 f161 f162 f163 f164 f165 f166 f167 f168 f169 f170 f171 f172 f173 f174 f175 f176 f177 f178 f179 f180 f181 f182 f183 f184 f185 f186 f187 f188 f189 f190 f191 f192 f193 f194 f195 f196 f197 f198 f199 f200 f201 f202 f203 f204 f205 f206 f207 f208 f209 f210 f211 f212 f213 f214 f215 f216 f217 f218 f219 f220 f221 f222 f223 f224 f225 f226 f227 f228 f229 f230 f231 f232 f233 f234 f235 f236 f237 f238 f239 f240 f241 f242 f243 f244 f245 f246 f247 f248 f249 f250 f251 f252 f253 f254 f255 f256 f257 f258 f259 f260 f261 f262 f263 f264 f265 f266 f267 f268 f269 f270 f271 f272 f273 f274 f275 f276 f277 f278].

#### Features Extraction Performance Comparison

Features extraction performance is based on the comparison of some other well-known features extraction methods, such as Histogram of Oriented Gradients (HOG) and Speeded Up Robust (SURF) features extraction. The comparison is performed by comparing the computational time of feature extraction and the correct recognition percentage of the extracted features extraction methods. A total of 1000 image samples are used to determine the features extraction performance comparison. Table 3 shows the comparison of features extraction method.

### 3.4. Recognition of the Road Sign

After the feature extraction, the feature vector passes through to the artificial neural network (ANN) for the recognition task. The ANN is very reliable and efficient for pattern recognition. The explanation of artificial neural network design, initialization of parameters, training network, validation network, test image and implementation are as follows.

#### 3.4.1. Artificial Neural Network Design

**Figure 9:**
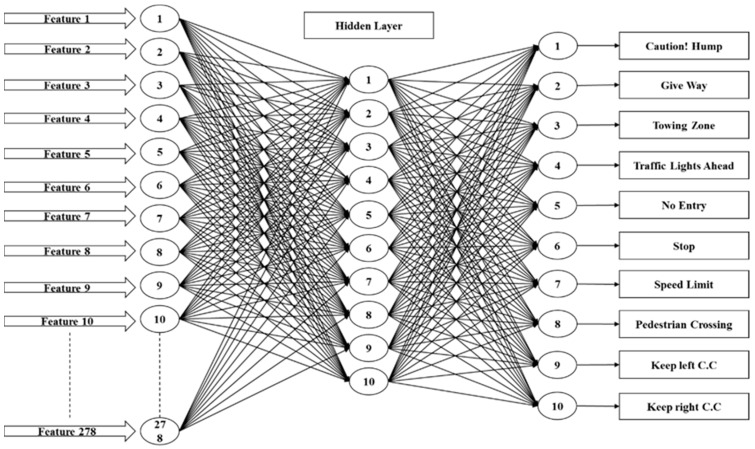
Neural network architecture.

An artificial neural network is implemented by using the Neural Network Pattern Recognition Tool in MATLAB. The standard network that is used for pattern recognition, is a two-layer feed-forward network with a sigmoid transfer function in the hidden layer, and a softmax transfer function in the output layer. For this system the number of hidden neurons is set to 10, which is more efficient and more reliable for this proposed system. The number of output neurons is set to 10, which is equal to the number of elements in the target vector (the number of sign categories). For the training of the multiple layer ANN, a systematic method is applied with back propagation-learning algorithm to the network. The main objective of the training is to adjust the weight so that the input produces desired output, and the neural network architecture is shown in Figure 9.

#### 3.4.2. Training Data Collection

**Figure 10:**
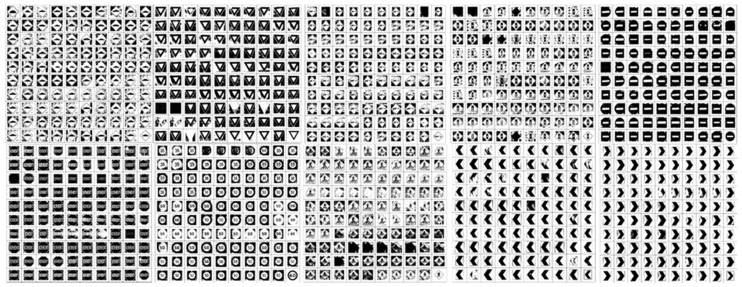
Training, testing, and validation image set for neural network.

The proposed system has used 10 specific Malaysian road signs and 100 training samples for each type of road sign to train the network to recognize those signs properly. Training images are collected by acquiring appropriate frames from video sequences, which were captured on different roads and highways in Malaysia in a real-time environment from 8:00 a.m. to 6:00 p.m. These 10 types of road signs were selected because of the different shapes, colors, and pictograms that were available for those road signs. If this proposed system can correctly classify all those signs, other signs can also be classified. From a total of 1000 samples, the neural network randomly selects 700 training samples, 150 test and 150 validation samples by default. Examples of training sets as well as test and validation sets of images are shown in Figure 10.

## 4. Experimental Results

The experiment may take place in number of steps. An Intel Core-i5 2.50 GHz CPU computer with 4 GB of RAM is used to run this program to recognize road signs. The prototype is developed within the MATLAB environment. The image processing toolbox, computer vision toolbox and neural network toolbox are used to implement this system.

A digital camera was mounted on the dashboard of a moving vehicle to capture video from a real-time environment. This video is segmented frame by frame with 1 s intervals, and it went through a hybrid color segmentation algorithm to identify the available or not road sign candidates. This hybrid color segmentation algorithm contains a RGB histogram equalization, RGB color segmentation, modified grey scale segmentation, binary image segmentation and shape matching algorithm. This hybrid color algorithm determines the exact position and properties of the target road sign. Then, according to that position and properties, the target road sign is extracted. At this point, there is no valuable information loss because the target road sign is extracted from the original image frame. This extracted image is converted into a grayscale image and normalized to 128-by-128 pixels. The normalized image is smoothened by a noise removal algorithm and it is converted into a binary image. This candidate image passes through the feature extraction process to extract the 278 feature vector. This feature vector is used to train the artificial neural network for recognition of the road sign.

### 4.1. Training, Testing, and Validation Data

To get an efficient response from the network, it is necessary to have a reasonable number of training samples. For this proposed system, a set of 100 samples is used for each class of road sign and 1000 samples for 10 classes of road sign. The extracted 1000-by-278 features vector is used as a ANN input data set for training, testing, and validating the network.

**Figure 11:**
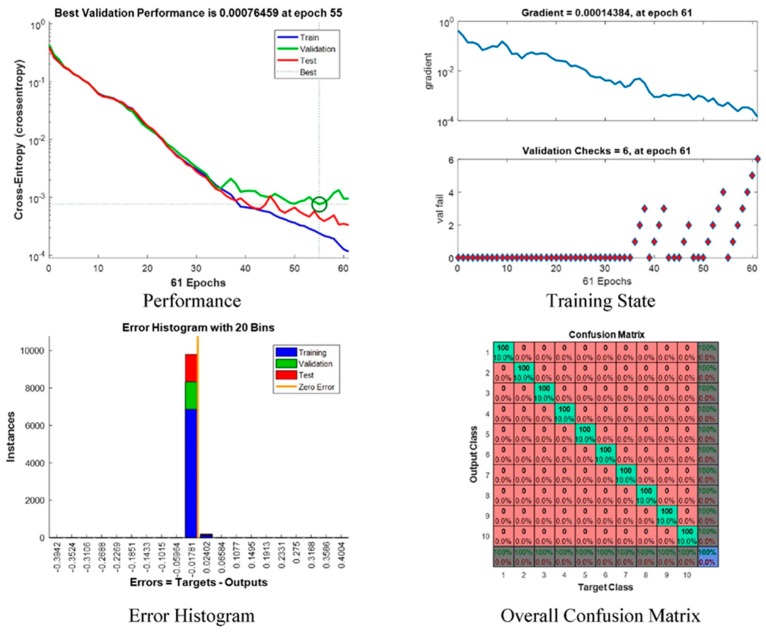
Neural Network: Performance, Training State, Error Histogram, and Overall Confusion Matrix.

Figure 11 shows the neural network’s performance, training state, error histogram, and overall confusion matrix. From the performance plot, the cross-entropy error is maximum at the beginning of training. For this proposed system, the best validation performance is at epoch 55, and at this point the cross-entropy error is very close to zero. On the training state plot, the maximum validation check 6 at epoch 61 and at this point, the neural network halts the training process to give best performance. The error histogram plot represents that the error of this proposed system is very close to zero. An overall confusion matrix is three sets of combined confusion matrices, which are the training confusion matrix, validation confusion matrix, and testing confusion matrix. This overall confusion matrix plot shows 100% correct classification for this proposed system.

**Figure 12:**
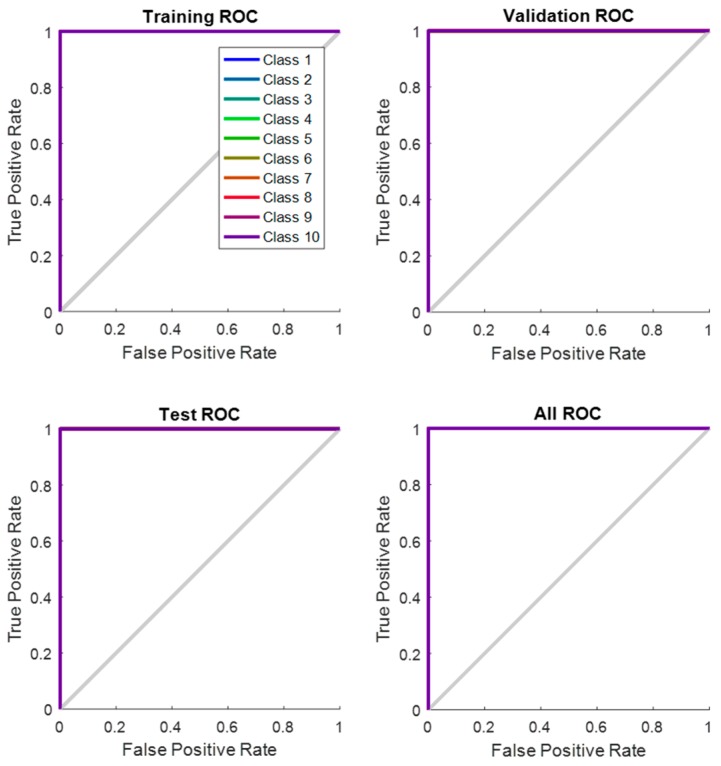
Network Receiver Operating Characteristic (ROC).

Receiver Operating Characteristic (ROC) curve of the network which illustrates true positive rate verses false positive rate at various threshold settings of the network, is shown in Figure 12. Area under the curve (AUC) shows a maximum perfect result for this proposed system. At the neural network train, test and validation conclusion, this network performs 100% correct classification of 10 classes of road sign.

### 4.2. Experiment with Malaysian Traffic Sign Database

Traffic signs are set up along the roadside, as an indication to instruct a driver to obey some traffic regulation. Some traffic signs are used to indicate a potential danger. There are two different groups of traffic signs in Malaysia: ideogram-based and text-based sign. Ideogram-base traffic signs use simple ideographs to express the meaning while the text-based traffic sign expression contains text with other symbols such as arrows.

**Figure 13:**
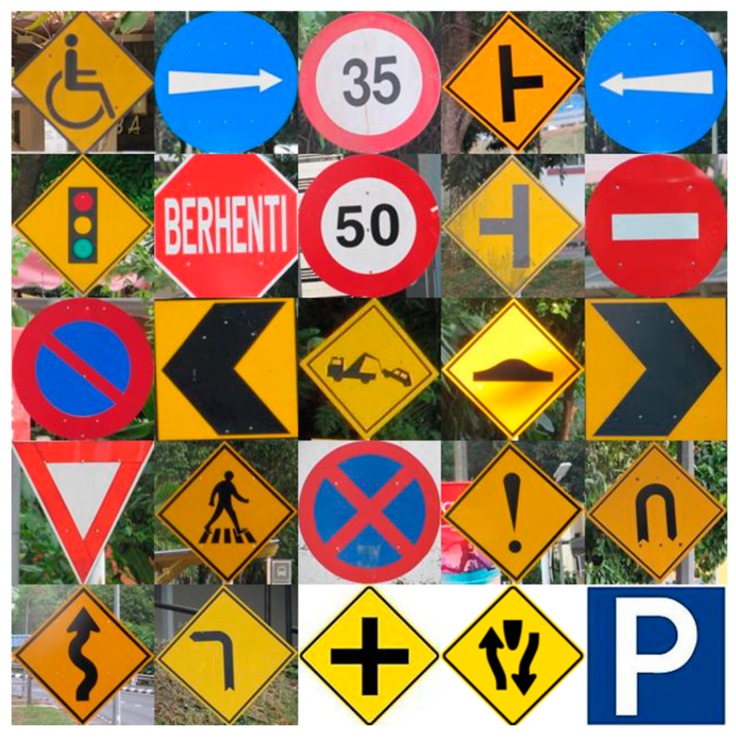
Malaysian traffic signs dataset example.

**Figure 14:**
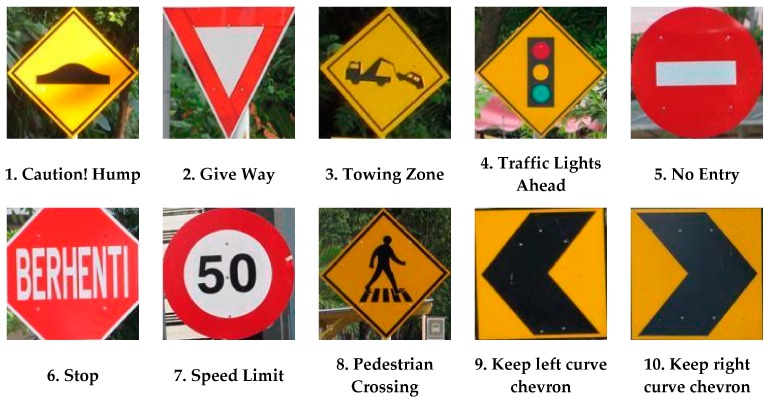
Ten class of traffic sign acquired from the Malaysian traffic sign database.

The Malaysian traffic sign database [64,65] consists of 100 classes of traffic sign used in Malaysia. Some examples have been shown in Figure 13. From this database, 10 classes of traffic sign are extracted as proposed for this system are shown in Figure 14.

**Figure 15:**
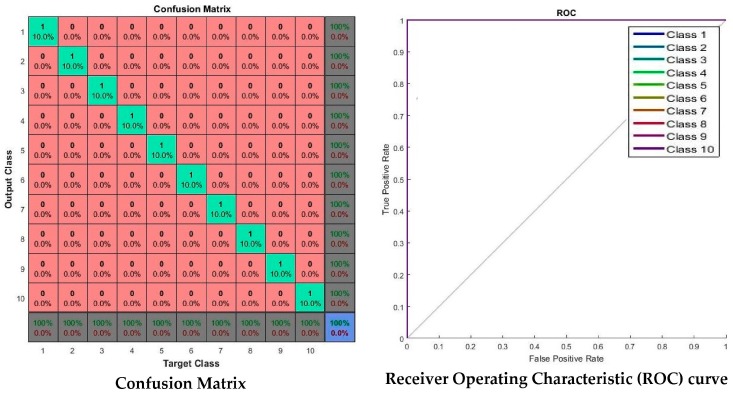
Malaysian Traffic Sign Database: Confusion Matrix, and Receiver Operating Characteristic (ROC).

Figure 15 is the experimental result with Malaysian traffic sign database which shows that all 10 classes of road signs are correctly classified and there are no misclassifications. The Receiver Operating Characteristic (ROC) curve shows all classes of road signs achieved the maximum area under the curve (AUC) and it shows a maximum perfect result for the Malaysian Traffic Sign Database.

### 4.3. Experiment with LISA Dataset

The LISA dataset [66] contains a large number of United States (US) traffic signs. The LISA dataset covers 47 type of US traffic signs, with a total of 7855 annotations on 6610 frames. The sign image sizes vary from 6-by-6 to 167-by-168 pixels and the full image frame sizes vary from 640-by-480 to 1024-by-522 pixels. To conduct the experiment with the LISA dataset, 20 class of US traffic sign image samples are taken into consideration because they are also commonly used in Malaysia. The extracted sign type and number of extracted signs are listed in Table 4.

A total of 1371 US traffic signs are used to evaluate the proposed methodology. The performance result is shown in Table 5.

**Figure 16:**
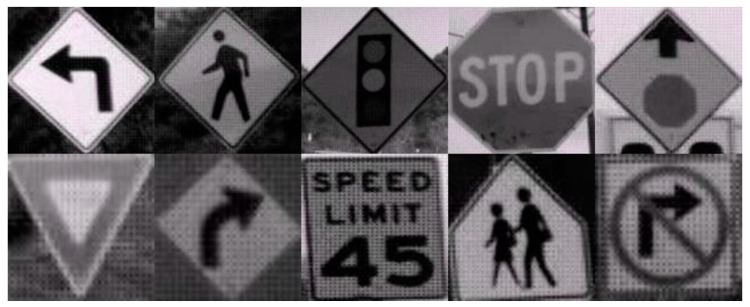
LISA dataset example.

Table 5 shows that the training number 5 has a better result because it has the lower error percentage (8.75273 e^−1^) then the other four training methods. This training number is considered for evaluating the performance. The performance is evaluated based on the confusion matrix and receiver operating characteristic (ROC) curve. Figure 16 shows some examples of LISA traffic signs.

**Figure 17:**
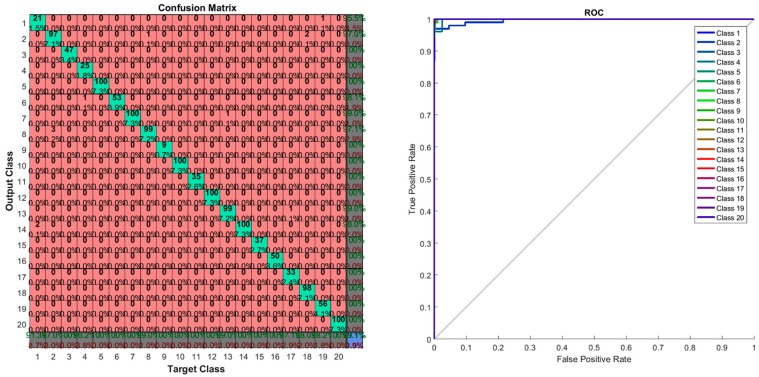
Confusion matrix and ROC curve of the LISA dataset experiment.

The confusion matrix and ROC curve are shown in Figure 17. In the confusion matrix, the red cubes present the incorrect classification and the green cubes present the correct classification according to the output class and target class. On the right bottom corner, the blue cube shows the overall percentage of the classification which in this case is 99.10% of correct classification performance. The ROC curve shows the area under the curve of every (AUC) 20 class testing samples. All testing class samples have achieved the maximum area under curve (AUC) except a few testing class samples that are misclassified as other classes. On the ROC curve, the top left corner shows that a small amount of classes do not achieve the maximum area under curve (AUC). At the conclusion of the experiment with the LISA dataset, the proposed method gives the desired classification performance.

### 4.4. Experiment with Convolution Neural Network (CNN)

**Figure 18:**
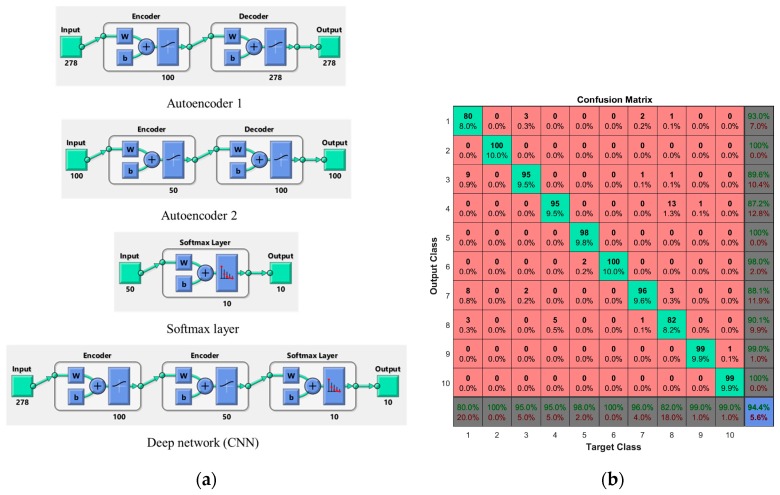
Experiment with CNN, (**a**) CNN architecture and (**b**) CNN confusion matrix.

The proposed method has been tested with a CNN which has two hidden layers to classify traffic signs. Firstly, the hidden layers have been trained individually in an unsupervised fashion using autoencoders. Then a final softmax layer has been trained, and joined with the two hidden layers to form a deep network, which is trained one final time in a supervised fashion. A total of 1000 real-time traffic sign samples are used to get the experimental results with CNN. Figure 18a shows the architecture of CNN and Figure 18b shows the CNN confusion matrix. The number in the bottom right-hand square of the confusion matrix gives the overall accuracy, which is 94.40%.

### 4.5. Experiment with Real-Time

**Figure 19:**
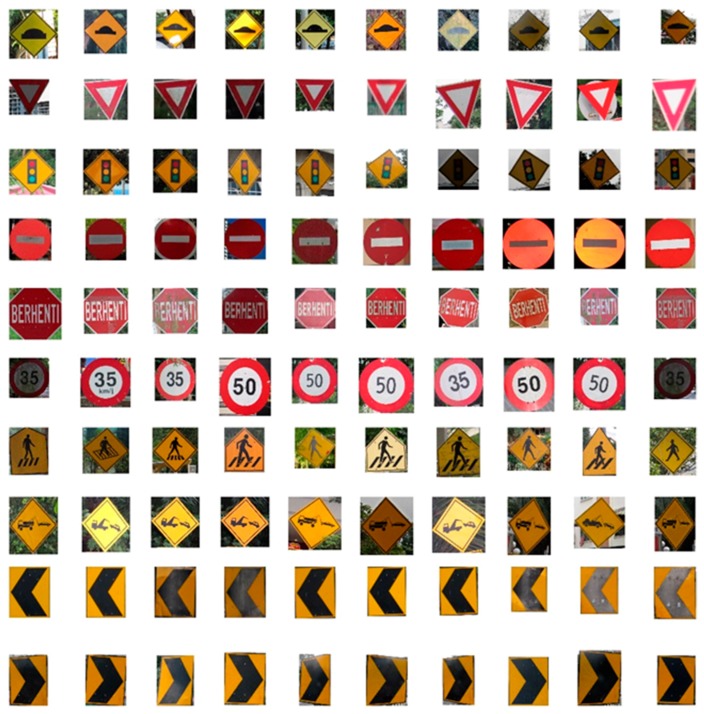
Example real-time input images.

Real-time test images are collected by acquiring targeted frames from a video sequence which is recorded from a real-time environment instantly as per Table 1. For this real-time experiment, 10 classes of sign are selected. Every class of sign contains 100 sample frames which are extracted from the video sequence, and in total 1000 sample frames are used to get real-time experimental results. These selected frames are then passed through the detection process, and the output image is a 128-by-128 binary image. This binary image is converted into a 128-by-128 binary matrix for the feature extraction process, and a 278 feature vector is extracted from each binary image. This feature vector is the input of the ANN to recognize which class of road sign it is. Figure 19 shows some examples of real-time input signs.

**Figure 20:**
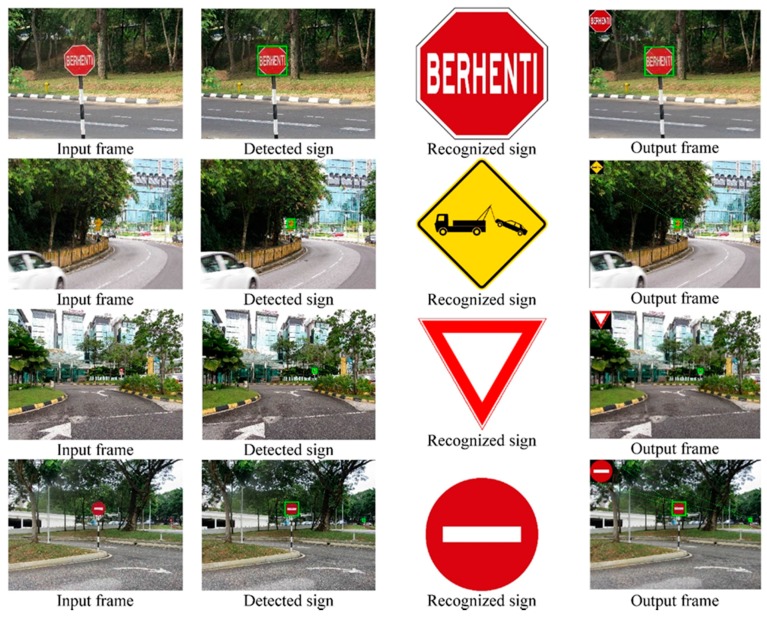
Some experimental input frames and the corresponding output frames.

Figure 20 shows real-time experimental results of “Stop” sign, “Towing zone” sign, “Yield” sign and “No entry” sign. The first column represents the input frames, the second column is the detected signs, the third column is the recognized signs and the fourth column represents the corresponding output frames.

**Figure 21:**
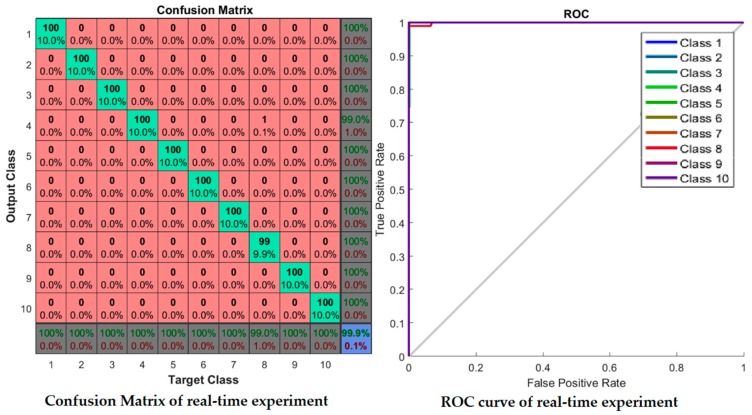
Confusion Matrix and ROC curve of a real-time experiment.

The real-time experiment confusion matrix and receiver operating characteristic (ROC) curve are shown in Figure 21. In this confusion matrix, the high numbers of correct responses are shown in the green squares. The low numbers of incorrect responses are shown in the red squares. The lower right blue square illustrates the overall accuracy. A class 8 sign, “Pedestrian Crossing”, was misclassified with a sign class 4, a “Traffic Lights Ahead” sign. The remaining other sign classes are correctly classified. The ROC curve shows that all classes of sign achieved the maximum area under curve (AUC) except class 8. A single misclassification of class 8 occurred due to the numerous (more than 10) non-standard “Pedestrian Crossing” sign formats that exist on actual Malaysian roadsides.

From the confusion matrix of the real-time experiment data, the evaluation parameters are precision, sensitivity, specificity, F-measure, false positive rate (*FPR*), and accuracy rate (*AR*) which are based on the number of true positive (*TP*), false positive (*FP*), true negative (*TN*), and false negative (*FN*) values as indicated in Table 6. True positive (*TP*) and true negative (*TN*) are defined as the traffic signs that are correctly recognized as the correct class and when other classes of traffic signs are correctly recognized as other class of traffic signs. False positive (*FP*) is defined as the traffic sign that is not recognized correctly. For the false negative (*FN*), a class of traffic sign is incorrectly recognized as another class of traffic sign.

In Table 7, a comparison between the proposed method and other existing methods based on the evaluation parameters is shown.

Table 8 presents the evaluated proposed system performance based on Neural Networks (NN). IDSIA [71] team used Committee of CNNs method to achieve a 99.46% of correct recognition rate, and this proposed system achieved a 99.90% correct recognition rate.

### 4.6. Classification Algorithm Performance

For the classification algorithm performance, the proposed input vector set is applied as an input vector set to the 23 different classification algorithms which are shown in Table 9. In these 23 classification algorithms, model number 1.23, which is the proposed classifier for this system, gives the best accuracy with 99.90%. Model 1.12 gives the nearest accuracy of 95.0% which is given by a Fine KNN classifier. Model 1.18 and model 1.22 which give the worst accuracy of 25%, are produced by the Boosted Trees and RUSBoosted Trees classifiers, respectively.

### 4.7. Robustness Testing

**Figure 22:**
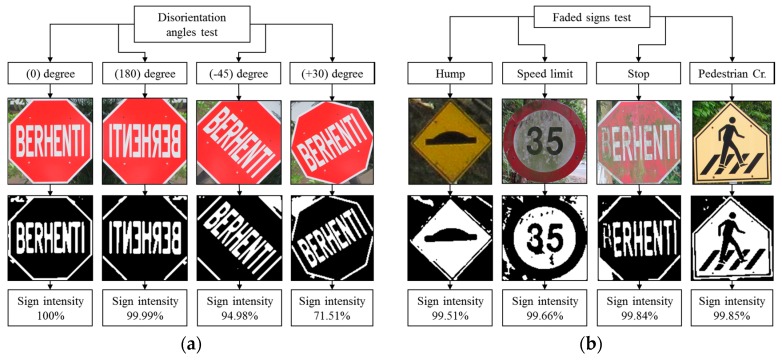
Robustness testing, (**a**) Disorientation angles test; (**b**) Faded signs test.

**Figure 23:**
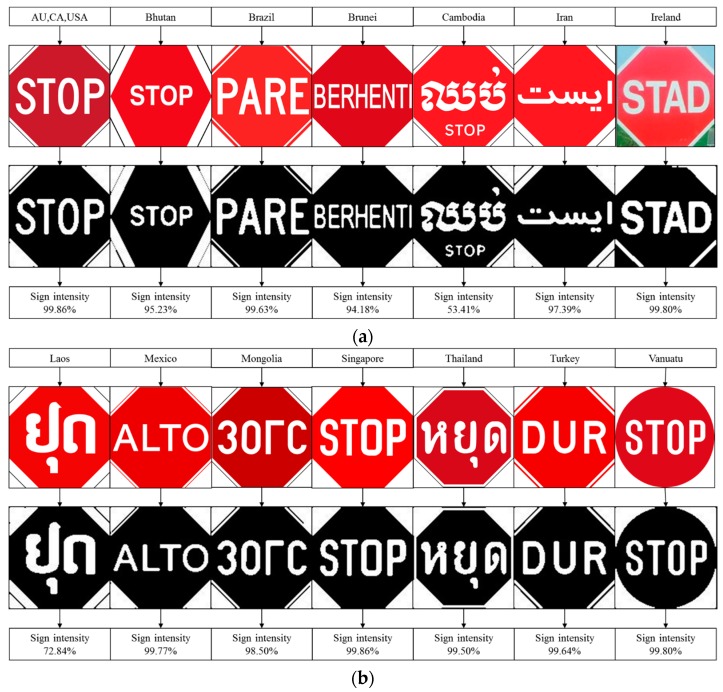
Robustness testing, (**a**) Seven set of different countries’ stop sign; (**b**) Seven sets of different countries’ stop signs.

**Figure 24:**
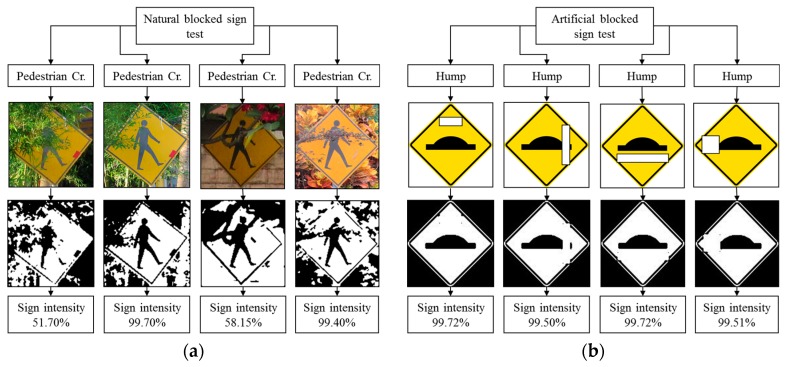
Robustness testing. (**a**) Natural blocked sign test; and (**b**) Artificial blocked sign test.

**Figure 25:**
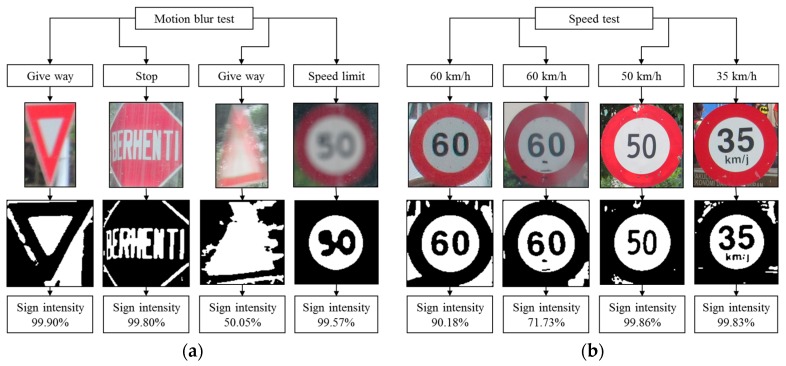
Robustness testing. (**a**) Motion blur test, and (**b**) Speed test.

**Figure 26:**
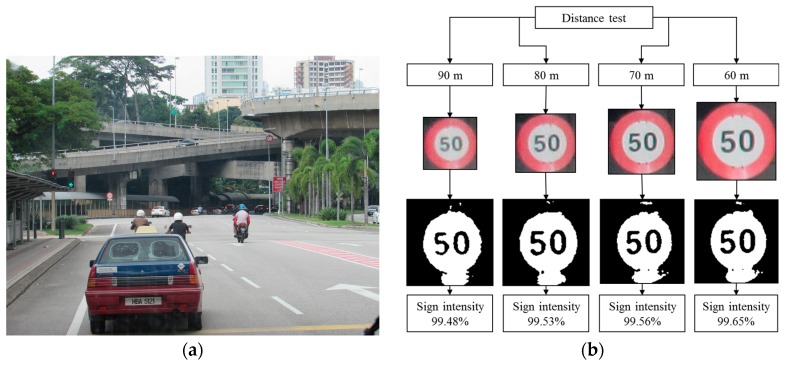
Robustness testing. (**a**) Original image frame; (**b**) Distance test.

Robustness testing is a testing methodology used to detect the vulnerabilities of a component under unexpected inputs or in a stressful environment. For this proposed system, some of the robustness testing was carried out with natural images and some are synthetic images. Figure 22, Figure 23, Figure 24, Figure 25 and Figure 26 illustrate the robustness testing of the proposed system.

### 4.8. Summary of Experiment Results

As a summary of our experimental results, the proposed method has been tested with a Malaysian traffic sign database [64,65], a real-time Malaysian database (with CNN and with ANN), and the LISA dataset [66]. Confusion matrixes and ROC curves are used to evaluate the classification performance. Different features extraction and their results have also been discussed earlier. Table 10 shows the summary of the experimental results.

## 5. Conclusions

Road and traffic sign recognition is a field of study that can be used to aid the development of intelligent transportation systems or car advisory systems. It continuously monitors the driver, vehicle and road in order, for example, to inform the driver in time of upcoming decision points, regarding navigation and potentially risk traffic situations. The aim of this research was to overcome the current limitations of real-time road sign recognition systems, such as single-color or single-class, specific country, and non-standard road signs. The proposed method is developed with a detection and a recognition stage. Detection is performed by capturing video frames with a dashboard camera in real-time from the highway. That frame goes through a hybrid color segmentation algorithm to identify the availability of the road sign candidates. For the first time in a road sign recognition system, a robust custom features extraction method was introduced to extract multiple features from a single image. This robust custom features an extraction process is based on a size independent method, and obtained 278 features for a single sign. This feature vector goes through a pre-trained ANN for recognition of road signs. ANN learning was performed with 100 sample images for each class of road sign, or a total 1000 sample images for 10 classes of road sign. The recognition performance is evaluated by using confusion matrix analysis, with two different Malaysian traffic sign datasets which are a standard dataset and a real-time dataset and one publicly available dataset (LISA). Results show that the algorithm achieved 100% accuracy with the Malaysian traffic sign dataset, 99.10% accuracy with the LISA dataset and an average of 99.90% accuracy with 99.90% of sensitivity, 0.001 of false positive rate and 0.33 s of processing time, with a real-time Malaysian traffic sign dataset. The experimental results are compared with existing methods and classifiers, showing the correctness of the proposed method. Additionally robustness testing shows that this proposed system is robust. The main limitation of this proposed system is that signs obscured by other vehicles or trees may not be recognized.

## Figures and Tables

**Table 1:** Environmental condition for image acquisition.

Environment	Real-Time
Weather	Rainy, sunny, cloudy
Video capturing time	8 a.m. to 6 p.m.
Background	Complex; not fixed
Frame rate	29 f/s
Horizontal field-of-view	Approximately 75°
Video frame width	1920
Video frame height	1080
Data rate	30,583 kbps
Maximum vehicle speed	65 km/h
Average vehicle speed	55 km/h
Maximum distance between sign and camera	90 m
Total number of frame acquired	17,400
Traffic sign condition	Standard, non-standard
Traffic sign type	1. Caution! Hump, 2. Give Way, 3. Towing Zone, 4. Traffic Lights Ahead, 5. No Entry, 6. Stop, 7. Speed Limit, 8. Pedestrian Crossing, 9. Keep left curve chevron sign, 10. Keep right curve chevron sign
Number of signs acquired per class	100
Total number of signs acquired	1000

**Table 2:** Submatrix conditions.

$S_{11}=\sum_{m=1}^{32}\sum_{n=1}^{32}A_{mn}$	$S_{12}=\sum_{m=1}^{32}\sum_{n=33}^{64}A_{mn}$	$S_{13}=\sum_{m=1}^{32}\sum_{n=65}^{96}A_{mn}$	$S_{14}=\sum_{m=1}^{32}\sum_{n=97}^{128}A_{mn}$
$S_{21}=\sum_{m=33}^{64}\sum_{n=1}^{32}A_{mn}$	$S_{22}=\sum_{m=33}^{64}\sum_{n=33}^{64}A_{mn}$	$S_{23}=\sum_{m=33}^{64}\sum_{n=65}^{96}A_{mn}$	$S_{24}=\sum_{m=33}^{64}\sum_{n=97}^{128}A_{mn}$
$S_{31}=\sum_{m=65}^{96}\sum_{n=1}^{32}A_{mn}$	$S_{32}=\sum_{m=65}^{96}\sum_{n=33}^{64}A_{mn}$	$S_{33}=\sum_{m=65}^{96}\sum_{n=65}^{96}A_{mn}$	$S_{34}=\sum_{m=65}^{96}\sum_{n=97}^{128}A_{mn}$
$S_{41}=\sum_{m=97}^{128}\sum_{n=1}^{32}A_{mn}$	$S_{42}=\sum_{m=97}^{128}\sum_{n=33}^{64}A_{mn}$	$S_{43}=\sum_{m=97}^{128}\sum_{n=65}^{96}A_{mn}$	$S_{44}=\sum_{m=97}^{128}\sum_{n=97}^{128}A_{mn}$

**Table 3:** Features Extraction Performance.

Method	Processing Time (s)/Image	Recognition Accuracy (%)
HOG	0.45	98.75
SURF	0.79	99.00
Proposed	0.12	99.90

**Table 4:** Experiment samples from the LISA dataset.

Sign Type	Number of Signs	Sign Type	Number of Signs
1. Do Not Enter	23	11. DIP	35
2. Keep Right	100	12. Speed Limit	100
3. No Left Turn	47	13. Stop	100
4. No Right Turn	26	14. Stop Ahead	100
5. Pedestrian Crossing	100	15. Curve Left	37
6. Round About	53	16. Curve Right	50
7. School	100	17. Slow	34
8. Signal Ahead	100	18. Yield	100
9. Do Not Pass	9	19. Yield Ahead	57
10. Added Lane	100	20. Merge	100

**Table 5:** Performance result with the LISA dataset.

Number of Training	Iterations	Training Time	Performance	Gradient	Validation Checks	Results
						Error (%)
**1**	115	0:00:05	0.00546	0.0131	6	1.45878 × 10^−0^
**2**	74	0:00:03	0.00569	0.0256	6	1.89642 × 10^−0^
**3**	87	0:00:04	0.00283	0.0148	6	1.75054 × 10^−0^
**4**	84	0:00:04	0.00161	0.00414	6	1.23997 × 10^−0^
**5**	**105**	**0:00:05**	**0.00621**	**0.00104**	**6**	**8.75273 × 10^−1^**

**Table 6:** Evaluation parameters.

Evaluation Parameters	Mathematical Equation	Result
Precision or Positive Predictive Value PPV	$PPV=\frac{TP}{TP+FP}$	0.999
Sensitivity or Recall or True Positive Rate TPR	$TPR=\frac{TP}{TP+FN}$	0.999
Specificity or True Negative Ratio TNR	$TNR=\frac{TN}{TN+FP}$	0.999
F-measure	$\frac{2\;\times \;TP}{2\;\times \;TP+FP+FN}$	0.999
False Positive Rate FPR	$\frac{FP}{FP+TN}$	0.001
Accuracy	$\frac{TP+TN}{TP+FN+FP+TN}$	0.999

**Table 7:** Comparison between proposed method and others existing method.

Reference	Precision (%)	Recall (%)	Specificity (%)	F-Measure (%)	False Positive Rate	Overall Accuracy (%)	Processing Time (s)
[67]	41.03	34.15	-	-	0.26	93.60	-
[68]	96.51	92.97	-	-	0.13	90.27	0.35
[69]	88.75	81.35	-	-	0.85	97.60	
[70]	-	-	-	-	1.2	86.70	
[8]	98.21	89.43	-	-	0.009	95.71	0.43
**Proposed Method**	**99.90**	**99.90**	**99.90**	**99.90**	**0.001**	**99.90**	**0.33**

**Table 8:** Evaluate the system performance based on Neural Networks (NN).

Reference	Classifier	Correct Recognition Rate
[71]	CNN	99.46%
[72]	CNN	98.31%
[73]	ANN	91.48%
[65]	CNN	99.00%
[74]	ANN	93.45%
[75]	ANN	91.50%
[76]	ANN	98.00%
[77]	ANN	98.62%
**Proposed Method**	**ANN**	**99.90%**

**Table 9:** Classification algorithms performance.

Model Number	Name of Classifier	Accuracy
1.1	Complex Tree	84.0%
1.2	Medium Tree	84.0%
1.3	Simple Tree	80.0%
1.4	Linear Discriminant	80.5%
1.5	Quadratic Discriminant	87.0%
1.6	Linear SVM	92.0%
1.7	Quadratic SVM	92.0%
1.8	Cubic SVM	92.0%
1.9	Fine Gaussian SVM	82.5%
1.10	Medium Gaussian SVM	92.5%
1.11	Coarse Gaussian SVM	83.0%
**1.12**	**Fine KNN**	**95.0%**
1.13	Medium KNN	83.0%
1.14	Coarse KNN	52.0%
1.15	Cosine KNN	85.0%
1.16	Cubic KNN	80.5%
1.17	Weighted KNN	89.0%
**1.18**	**Boosted Trees**	**25.0%**
1.19	Bagged Trees	93.5%
1.20	Subspace Discriminant	93.0%
1.21	Subspace KNN	94.0%
**1.22**	**RUSBoosted Trees**	**25.0%**
**1.23**	**Proposed classifier**	**99.90%**

**Table 10:** Summary of experimental results.

Experiment	Database	Number of Test Samples	Features Extraction	Classifier	Classification Accuracy
**1**	Malaysian real-time	1000	HOG	ANN	98.75%
**2**	Malaysian real-time	1000	SURF	ANN	99.00%
**3**	Malaysian traffic sign	100	Proposed	ANN	100%
**4**	LISA	1371	Proposed	ANN	99.10%
**5**	Malaysian real-time	1000	Proposed	CNN	94.40%
**6**	Malaysian real-time	1000	Proposed	ANN	99.90%

## References

[1] Malik R., Khurshid J., Ahmad S.N. Road sign detection and recognition using colour segmentation, shape analysis and template matching. Proceedings of the 2007 International Conference on Machine Learning and Cybernetics.

[2] Min K.W., Choi J.D. Design and implementation of an intelligent vehicle system for autonomous valet parking service. Proceedings of the 2015 10th Asian Control Conference (ASCC).

[3] Khan A.M., FischerWolfarth J., Meyer G. (2014). Design of real-time transition from driving assistance to automation function: Bayesian artificial intelligence approach. Advanced Microsystems for Automotive Applications 2014: Smart Systems for Safe, Clean and Automated Vehicles.

[4] Yeshodara N.S., Nagojappa N.S., Kishore N. Cloud based self driving cars. Proceedings of the 2014 IEEE International Conference on Cloud Computing in Emerging Markets (CCEM).

[5] Hayashi E. A navigation system with a self-drive control for an autonomous robot in an indoor environment. Proceedings of the RO-MAN 2007—The 16th IEEE International Symposium on Robot and Human Interactive Communication.

[6] Zhang T., Lv J., Yang J. Road sign detection based on visual saliency and shape analysis. Proceedings of the 2013 IEEE International Conference on Image Processing.

[7] Chakraborty S., Deb K. Bangladeshi road sign detection based on ycbcr color model and dtbs vector. Proceedings of the 2015 International Conference on Computer and Information Engineering (ICCIE).

[8] Wali S.B., Hannan M.A., Hussain A., Samad S.A. (2015). An automatic traffic sign detection and recognition system based on colour segmentation, shape matching, and svm. Math. Probl. Eng..

[9] Rizvi R., Kalra S., Gosalia C., Rahnamayan S. Fuzzy adaptive cruise control system with speed sign detection capability. Proceedings of the 2014 IEEE International Conference on Fuzzy Systems.

[10] Liu H., Zhang L., Sun D., Wang D. (2015). Optimize the settings of variable speed limit system to improve the performance of freeway traffic. IEEE Trans. Intell. Transp. Syst..

[11] Li Y., Mogelmose A., Trivedi M.M. (2016). Pushing the “speed limit”: Towards high-accuracy U.S. Traffic sign recognition with convolutional neural networks. IEEE Trans. Intell. Veh..

[12] Lin Y.T., Chou T., Vinay M.S., Guo J.I. Algorithm derivation and its embedded system realization of speed limit detection for multiple countries. Proceedings of the 2016 IEEE International Symposium on Circuits and Systems (ISCAS).

[13] Biswas R., Fleyeh H., Mostakim M. Detection and classification of speed limit traffic signs. Proceedings of the 2014 World Congress on Computer Applications and Information Systems (WCCAIS).

[14] Houben S., Stallkamp J., Salmen J., Schlipsing M., Igel C. Detection of traffic signs in real-world images: The german traffic sign detection benchmark. Proceedings of the 2013 International Joint Conference on Neural Networks (IJCNN).

[15] Yamamoto J., Karungaru S., Terada K. Japanese road signs recognition using neural networks. Proceedings of the SICE Annual Conference.

[16] Stallkamp J., Schlipsing M., Salmen J., Igel C. (2012). Man vs. Computer: Benchmarking machine learning algorithms for traffic sign recognition. Neural Netw..

[17] Cireşan D., Meier U., Masci J., Schmidhuber J. A committee of neural networks for traffic sign classification. Proceedings of the 2011 International Joint Conference on Neural Networks (IJCNN).

[18] Manual On Traffic Control Devices Traffic Sign Applications. http://www.jkrbentong.gov.my/images/e-Perpustakaan/01_Rujukan_Teknikal/03_Teknikal_Jalan/ATJ/02_Traffic_Control/2B-85_Traffic_Signs_Applications.pdf..

[19] Yilmaz T., Foster R., Hao Y. (2010). Detecting vital signs with wearable wireless sensors. Sensors.

[20] Carrasco J.-P., de la Escalera A.D.L.E., Armingol J.M. (2012). Recognition stage for a speed supervisor based on road sign detection. Sensors.

[21] Chen L., Li Q., Li M., Zhang L., Mao Q. (2012). Design of a multi-sensor cooperation travel environment perception system for autonomous vehicle. Sensors.

[22] García-Garrido M.A., Ocaña M., Llorca D.F., Arroyo E., Pozuelo J., Gavilán M. (2012). Complete vision-based traffic sign recognition supported by an i2v communication system. Sensors.

[23] Son S., Baek Y. (2015). Design and implementation of real-time vehicular camera for driver assistance and traffic congestion estimation. Sensors.

[24] Hoang T., Hong H., Vokhidov H., Park K. (2016). Road lane detection by discriminating dashed and solid road lanes using a visible light camera sensor. Sensors.

[25] Llorca D.F., Sánchez S., Ocaña M., Sotelo M.A. (2010). Vision-based traffic data collection sensor for automotive applications. Sensors.

[26] Wu J., Si M., Tan F., Gu C. Real-time automatic road sign detection. Proceedings of the Fifth International Conference on Image and Graphics (ICIG’09).

[27] Belaroussi R., Foucher P., Tarel J.P., Soheilian B., Charbonnier P., Paparoditis N. Road sign detection in images: A case study. Proceedings of the 2010 20th International Conference on Pattern Recognition (ICPR).

[28] Aliane N., Fernandez J., Mata M., Bemposta S. (2014). A system for traffic violation detection. Sensors.

[29] Shoba E., Suruliandi A. Performance analysis on road sign detection, extraction and recognition techniques. Proceedings of the 2013 International Conference on Circuits, Power and Computing Technologies (ICCPCT).

[30] Lin C.-C., Wang M.-S. (2012). Road sign recognition with fuzzy adaptive pre-processing models. Sensors.

[31] Paclík P., Novovicová J., Duin R.P. (2006). Building road-sign classifiers using a trainable similarity measure. IEEE Trans. Intell. Transp. Syst..

[32] Khan J.F., Bhuiyan S.M.A., Adhami R.R. (2011). Image segmentation and shape analysis for road-sign detection. IEEE Trans. Intell. Transp. Syst..

[33] Kumaraswamy R., Prabhu L.V., Suchithra K., Pai P.S.S., Abraham A., Mauri J.L., Buford J.F., Suzuki J., Thampi S.M. (2011). Svm based classification of traffic signs for realtime embedded platform. Advances in Computing and Communications, pt 4.

[34] Mariut F., Fosalau C., Avila M., Petrisor D. Detection and recognition of traffic signs using gabor filters. Proceedings of the 2011 34th International Conference on Telecommunications and Signal Processing (Tsp).

[35] Wahyono, Jo K.H. Information retrieval of led text on electronic road sign for driver-assistance system using spatial-based feature and nearest cluster neighbor classifier. Proceedings of the 2014 IEEE 17th International Conference on Intelligent Transportation Systems (Itsc).

[36] Zhang Q.S., Kamata S. (2013). Improved color barycenter model and its separation for road sign detection. IEICE Trans. Inf. Syst..

[37] Saponara S., Kehtarnavaz N., Carlsohn M.F. (2013). Real-time color/shape-based traffic signs acquisition and recognition system. Real-Time Image and Video Processing 2013.

[38] Kim J.B. (2013). Detection of traffic signs based on eigen-color model and saliency model in driver assistance systems. Int. J. Automot. Technol..

[39] Song L., Liu Z.Y. Color-based traffic sign detection. Proceedings of the 2012 International Conference on Quality, Reliability, Risk, Maintenance, and Safety Engineering (Icqr2mse).

[40] Qun C., Wang J., Wei L. Road sign detection using specific color-pair information. Proceedings of the 2012 International Conference on Machine Learning and Cybernetics.

[41] Kim S., Kim S., Uh Y., Byun H. (2012). Color and shape feature-based detection of speed sign in real-time. Proceedings of the 2012 IEEE International Conference on Systems, Man, and Cybernetics.

[42] Eom T.J., Goswami K., Kim B.G., Lee J. Hybrid color space based road sign detection technique. Proceedings of the 2012 IEEE International Conference on Consumer Electronics—Berlin (ICCE-Berlin).

[43] Zhang Q.S., Kamata S., Jusoff K., Xie Y. (2010). Pixel color feature enhancement for road signs detection. Second International Conference on Digital Image Processing.

[44] De La Escalera A., Moreno L.E., Salichs M.A., Armingol J.M. (1997). Road traffic sign detection and classification. IEEE Trans. Ind. Electr..

[45] Lauziere Y.B., Gingras D., Ferrie F.P. A model-based road sign identification system. Proceedings of the 2001 IEEE Computer Society Conference on Computer Vision and Pattern Recognition.

[46] Fleyeh H. (2006). Shadow and Highlight Invariant Colour Segmentation Algorithm for Traffic Signs.

[47] Lorsakul A., Suthakorn J. Traffic sign recognition for intelligent vehicle/driver assistance system using neural network on opencv. Proceedings of the 4th International Conference on Ubiquitous Robots and Ambient Intelligence.

[48] Ruta A., Li Y., Liu X. (2010). Real-time traffic sign recognition from video by class-specific discriminative features. Pattern Recogn..

[49] Joshi M., Singh M.J., Dalela S. Automatic colored traffic sign detection using optoelectronic correlation architectures. Proceedings of the 2008 IEEE International Conference on Vehicular Electronics and Safety.

[50] Deshmukh V.R., Patnaik G., Patil M. (2013). Real-time traffic sign recognition system based on colour image segmentation. Int. J. Comput. Appl..

[51] Mammeri A., Khiari E.-H., Boukerche A. Road-sign text recognition architecture for intelligent transportation systems. Proceedings of the 2014 IEEE 80th Vehicular Technology Conference (Vtc Fall).

[52] Greenhalgh J., Mirmehdi M. (2015). Recognizing text-based traffic signs. IEEE Trans. Intell. Transp. Syst..

[53] De La Escalera A., Armingol J.M., Pastor J.M., Rodríguez F.J. (2004). Visual sign information extraction and identification by deformable models for intelligent vehicles. IEEE Trans. Intell. Transp. Syst..

[54] Gil-Jimenez P., Gomez-Moreno H., Siegmann P., Lafuente-Arroyo S., Maldonado-Bascon S. Traffic sign shape classification based on support vector machines and the fft of the signature of blobs. Proceedings of the 2007 IEEE Intelligent Vehicles Symposium.

[55] Ai C., Tsai Y.J. (2011). Hybrid active contour–incorporated sign detection algorithm. J. Comput. Civil Eng..

[56] Ai C., Tsai Y. (2014). Geometry preserving active polygon-incorporated sign detection algorithm. J. Comput. Civil Eng..

[57] Balali V., Golparvar-Fard M. (2014). Video-based detection and classification of us traffic signs and mile markers using color candidate extraction and feature-based recognition. Computing in Civil and Building Engineering (2014).

[58] Balali V., Rad A.A., Golparvar-Fard M. (2015). Detection, classification, and mapping of us traffic signs using google street view images for roadway inventory management. Vis. Eng..

[59] Chen T., Lu S. (2016). Accurate and efficient traffic sign detection using discriminative adaboost and support vector regression. IEEE Trans. Veh. Technol..

[60] Gomez-Moreno H., Maldonado-Bascon S., Gil-Jimenez P., Lafuente-Arroyo S. (2010). Goal evaluation of segmentation algorithms for traffic sign recognition. IEEE Trans. Intell. Transp. Syst..

[61] Ali N.M., Sobran N.M.M., Ab Shukur S.A., Ghazaly M.M., Ibrahim A.F.T. Individual processing speed analysis for traffic sign detection and recognition. Proceedings of the 2013 IEEE International Conference on Smart Instrumentation, Measurement and Applications (ICSIMA 2013).

[62] Fleyeh H. Color detection and segmentation for road and traffic signs. Proceedings of the 2004 IEEE Conference on Cybernetics and Intelligent Systems.

[63] Shadeed W.G., Abu-Al-Nadi D.I., Mismar M.J. Road traffic sign detection in color images. Proceedings of the 2003 10th IEEE International Conference on Electronics, Circuits and Systems (ICECS2003).

[64] Lim K.H., Seng K.P., Ang L.-M. (2012). Mimo lyapunov theory-based rbf neural classifier for traffic sign recognition. Appl. Comput. Intell. Soft Comput..

[65] Lau M.M., Lim K.H., Gopalai A.A. Malaysia traffic sign recognition with convolutional neural network. Proceedings of the 2015 IEEE International Conference on Digital Signal Processing (DSP).

[66] Mogelmose A., Trivedi M.M., Moeslund T.B. (2012). Vision-based traffic sign detection and analysis for intelligent driver assistance systems: Perspectives and survey. IEEE Trans. Intell. Transp. Syst..

[67] Maldonado-Bascon S., Lafuente-Arroyo S., Gil-Jimenez P., Gomez-Moreno H., Lopez-Ferreras F. (2007). Road-sign detection and recognition based on support vector machines. IEEE Trans. Intell. Transp. Syst..

[68] García-Garrido M.A., Ocaña M., Llorca D.F., Sotelo M.A., Arroyo E., Llamazares A. Robust traffic signs detection by means of vision and v2i communications. Proceedings of the 2011 14th International IEEE Conference on Intelligent Transportation Systems (ITSC).

[69] Greenhalgh J., Mirmehdi M. (2012). Real-time detection and recognition of road traffic signs. IEEE Trans. Intell. Transp. Syst..

[70] Bui-Minh T., Ghita O., Whelan P.F., Hoang T. A robust algorithm for detection and classification of traffic signs in video data. Proceedings of the 2012 International Conference on Control, Automation and Information Sciences (ICCAIS).

[71] Cireşan D., Meier U., Masci J., Schmidhuber J. (2012). Multi-column deep neural network for traffic sign classification. Neural Netw..

[72] Sermanet P., LeCun Y. Traffic sign recognition with multi-scale convolutional networks. Proceedings of the 2011 International Joint Conference on Neural Networks (IJCNN).

[73] Rahman M.O., Mousumi F.A., Scavino E., Hussain A., Basri H. Real time road sign recognition system using artificial neural networks for bengali textual information box. Proceedings of the 2008 International Symposium on Information Technology.

[74] Rouabeh H., Abdelmoula C., Masmoudi M. (2016). A novel neural network based method developed for digit recognition applied to automatic speed sign recognition. Int. J. Adv. Comput. Sci. Appl. (IJACSA).

[75] Miah M.B.A. (2015). A real time road sign recognition using neural network. Int. J. Comput. Appl..

[76] Saha S.K., Chakraborty D. (2012). Neural network based road sign recognition. Int. J. Comput. Appl..

[77] Yin S., Liu L., Guo Y., Wei S. (2015). Fast traffic sign recognition with a rotation invariant binary pattern based feature. Sensors.

